# Supplementary material for: CDK4/6 inhibition mitigates stem cell damage in a novel model for taxane‐induced alopecia
Source: EMBO Mol Med. 2019 Sep 12;11(10):e11031. doi: 10.15252/emmm.201911031 (PMC6783643; doi:10.15252/emmm.201911031)
Supplement: Supplementary file 1 — Appendix [file EMMM-11-e11031-s001.pdf]

Appendix

Figure S1 – Taxanes induce apoptosis and mitotic aberrations within the human hair follicle matrix and stem/progenitor-rich outer root sheath following drug washout **(Page 2)**

Figure S2 – CDK4/6 inhibition by palbociclib induces G1 arrest in human hair follicle matrix keratinocytes **(Page 4)**

Figure S3 – G1 arrest through CDK4/6 inhibition is reversible in the human hair follicle matrix and is not cytotoxic **(Page 6)**

Figure S4 – Hair follicles that do not restart DNA synthesis in the human hair matrix following palbociclib drug washout do not show paclitaxel cytotoxicity **(Page 7)**

Figure S5 – Hair cycle analysis following palbociclib and/or paclitaxel treatment **(Page 8)**

Figure S6 – Palbociclib blocks paclitaxel induced accumulation of pH3+ cells in the human hair follicle lower outer root sheath **(Page 9)**

Figure S7 – Hair follicles that show resumed DNA synthesis in the outer root sheath following palbociclib drug washout show an accumulation of pH3+ cells in response to paclitaxel **(Page 10)**

Fig S1

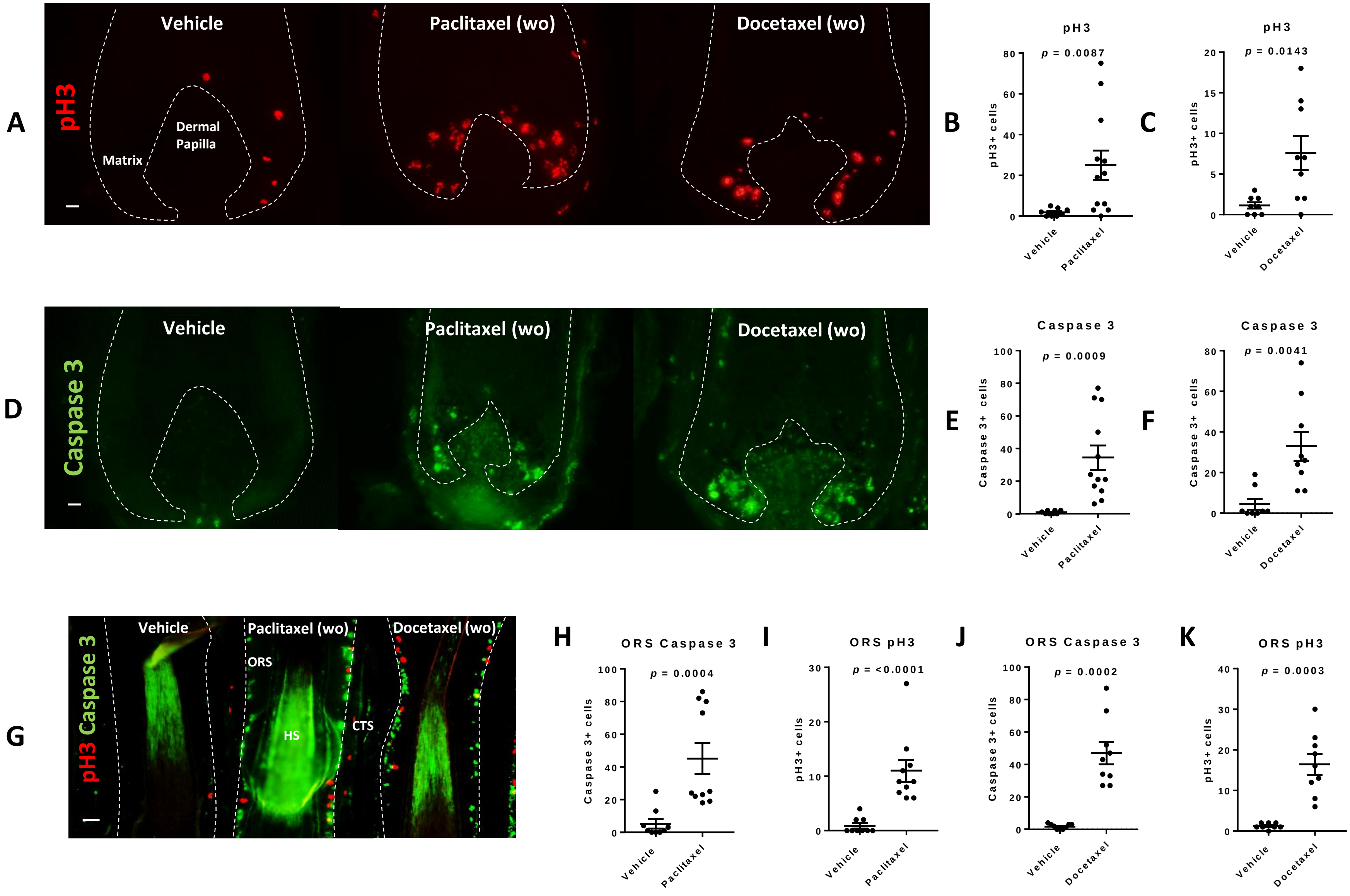

A
pH3
Vehicle
Paclitaxel (wo)
Docetaxel (wo)
Dermal
Papilla
Matrix
B
pH3
p = 0.0087
pH3+ cells
Vehicle
Paclitaxel
C
pH3
p = 0.0143
pH3+ cells
Vehicle
Docetaxel
D
Caspase 3
Vehicle
Paclitaxel (wo)
Docetaxel (wo)
E
Caspase 3
p = 0.0009
Caspase 3+ cells
Vehicle
Paclitaxel
F
Caspase 3
p = 0.0041
Caspase 3+ cells
Vehicle
Docetaxel
G
pH3 Caspase 3
Vehicle
Paclitaxel (wo)
Docetaxel (wo)
ORS
HS
CTS
H
ORS Caspase 3
p = 0.0004
Caspase 3+ cells
Vehicle
Paclitaxel
I
ORS pH3
p = <0.0001
pH3+ cells
Vehicle
Paclitaxel
J
ORS Caspase 3
p = 0.0002
Caspase 3+ cells
Vehicle
Docetaxel
K
ORS pH3
p = 0.0003
pH3+ cells
Vehicle
Docetaxel

Figure S1 – Taxanes induce apoptosis and mitotic aberrations within the human hair follicle matrix and stem/progenitor-rich outer root sheath following drug washout

A) Representative images of pH3 immunoreactivity in paclitaxel and docetaxel treated (24h) hair follicles (HFs) isolated following a subsequent drug washout period of 24-48h. 20 µm scale. **B-C)** Graphs showing increases in the number of pH3+ cells following paclitaxel and docetaxel treatment and washout in the hair matrix. Vehicle vs. paclitaxel – p = 0.0087 [**]; vehicle versus docetaxel – p = 0.0143 [*]. Welch's *t*-tests performed using *N* of 8-11 HFs from 3 patients. Data from "B**"** are utilised in main text Figure 6D.

D) Representative images of caspase 3 expression in paclitaxel and docetaxel treated (24h) HFs, isolated following a subsequent drug washout period of 24-48h. 20 µm scale. **E-F)** Graphs showing an increase in the number of caspase 3+ cells following paclitaxel and docetaxel treatment and washout in the hair matrix. Vehicle vs. paclitaxel – p = 0.0009 [***]. Vehicle versus docetaxel - p = 0.0041 [**]. Welch's *t*-tests performed using *N* of 8-12 HFs from 3 patients. Data from "E", are utilised in main text Figure 6C.

G) Representative images of accumulating caspase 3+ and pH3+ cells in the outer root sheath (ORS) of hair follicles treated with docetaxel and paclitaxel following drugwash out. 50 µm scale. **H-I)** Graphs showing increases in the number of caspase 3+ and pH3+ cells following paclitaxel treatment and washout in the ORS. p = 0.004 [***]) and $p < 0.0001$ [****] respectively. Mann Whitney *U* tests performed using *N* of 9-10 HFs from 3 patients. Data from "H-I", are utilised main text Figure 7C-D. **J-K)** Graphs showing increases in the number of caspase 3+ and pH3+ cells following docetaxel treatment and washout in the ORS. p = 0.0002 and 0.0003 respectively [***]. Welch's *t*-tests performed using *N* of 8-9 HFs from 3 patients. CTS – connective tissue sheath; HS = hair shaft; ORS – outer root sheath; wo – washout.

Fig S2

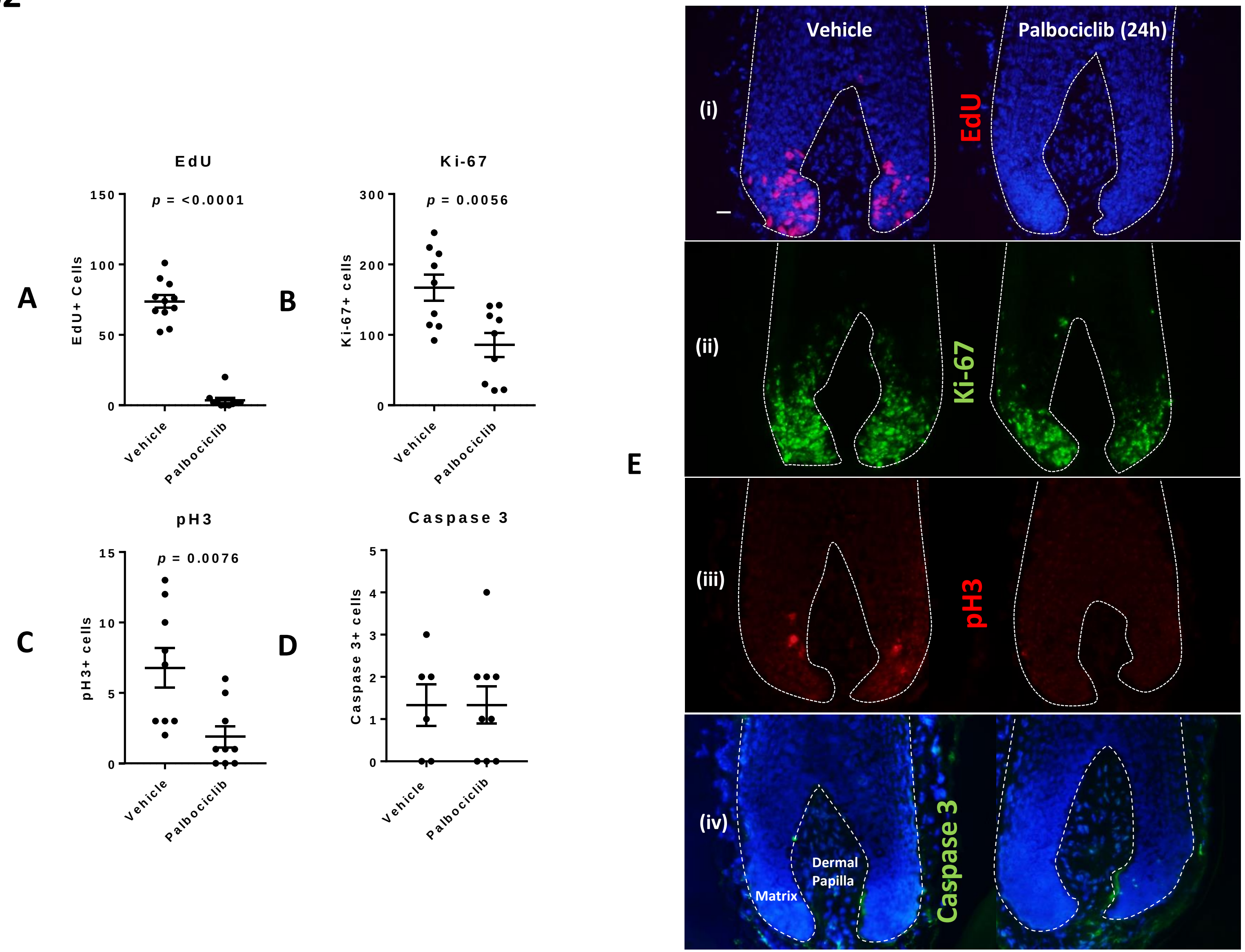

A
EdU
p = <0.0001
150
100
50
0
EdU+ Cells
Vehicle
Palbociclib
B
Ki-67
p = 0.0056
300
200
100
0
Ki-67+ cells
Vehicle
Palbociclib
C
pH3
p = 0.0076
15
10
5
0
pH3+ cells
Vehicle
Palbociclib
D
Caspase 3
5
4
3
2
1
0
Caspase 3+ cells
Vehicle
Palbociclib
E
Vehicle
Palbociclib (24h)
(i)
EdU
(ii)
Ki-67
(iii)
pH3
(iv)
Caspase 3
Dermal
Papilla
Matrix

Figure S2 – CDK4/6 inhibition by palbociclib induces G1 arrest in human hair follicle matrix keratinocytes

A) Treatment with the CDK4/6 inhibitor palbociclib (1 µM, 24h) induced a profound block in DNA synthesis as demonstrated by a significant decrease in the number of EdU+ keratinocytes in the hair matrix (p =<0.0001 [****]). Mann Whitney *U* test performed using *N* of 10-11 HFs from 3 patients.

B) Palbociclib significantly reduces the number of cycling Ki-67+ keratinocytes in the hair matrix (p = 0.0056 [**]). Unpaired *t*-test performed using *N* of 9 HFs from 3 patients.

C) Palbociclib significantly reduces the total number of mitotic pH3+ keratinocytes in the human hair matrix (p = 0.0076 [**]). Unpaired *t*-test performed using *N* of 9 HFs from 3 patients.

D) Palbociclib does not increase the number of cleaved caspase 3+ cells in the hair matrix. Mann Whitney *U* test performed using N of 6-9 HFs from 3 patients.

E) Immunofluorescence images representing how 1 µM palbociclib treatment influences (i) EdU incorporation; (ii) Ki-67 expression; (iii) pH3 immunoreactivity and (iv) cleaved caspase 3 immunoreactivity in the hair matrix after 24h.

Scales bars are 20 µm.

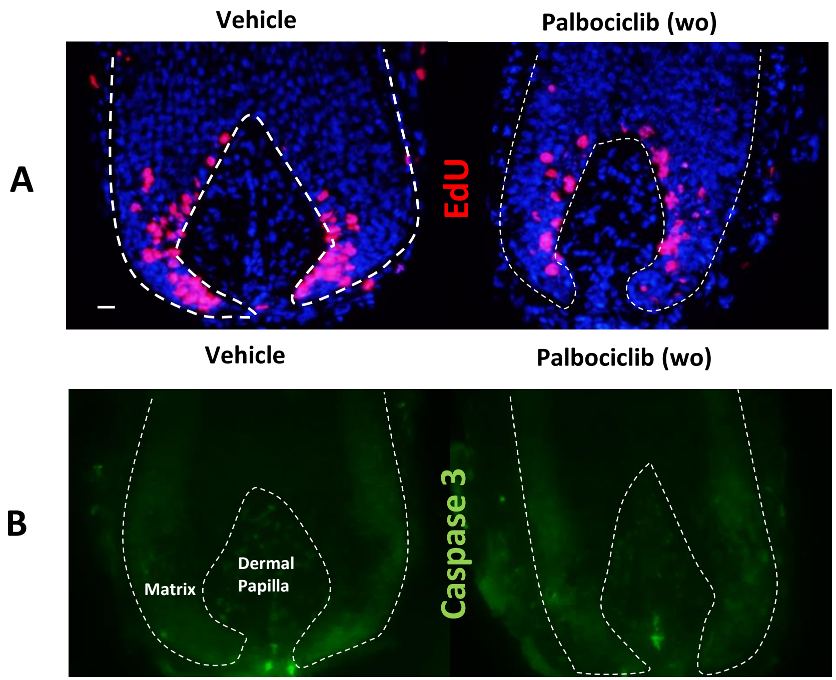

Figure S3 – G1 arrest through CDK4/6 inhibition is reversible in the human hair follicle matrix and is not cytotoxic

Representative fluorescence images of EdU incorporation **(A)** and cleaved caspase 3 expression **(B)** in the human hair follicle (HF) matrix in vehicle versus palbociclib-only treated HFs following drug washout. Data highlights that the G1 arrest induced by palbociclib is reversible and not cytotoxic. Images correspond to quantitative data featured in main text (see **Figure 6B;6C**). 20 µm scale.

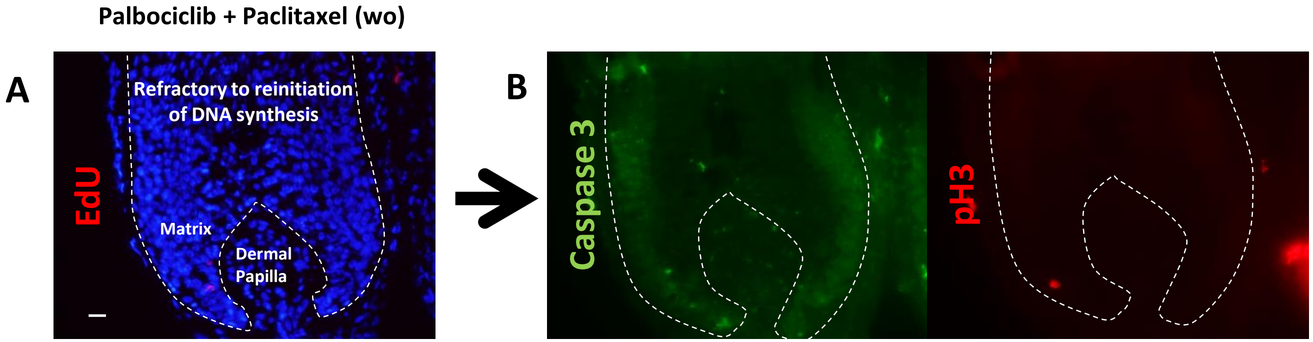

Figure S4 – Hair follicles that do not restart DNA synthesis in the human hair matrix following palbociclib drug washout do not show paclitaxel cytotoxicity

In most hair follicles (HFs) proliferation resumed following washout of palbociclib, which was met with paclitaxel cytotoxicity (see **Figure 6**). Where proliferation did not resume in a subset of HFs e.g in **(A)**, there was no corresponding increase in cleaved caspase 3 and pH3 **(B)**, further emphasising the cell cycle dependency of paclitaxel cytotoxicity. 20 µm scale.

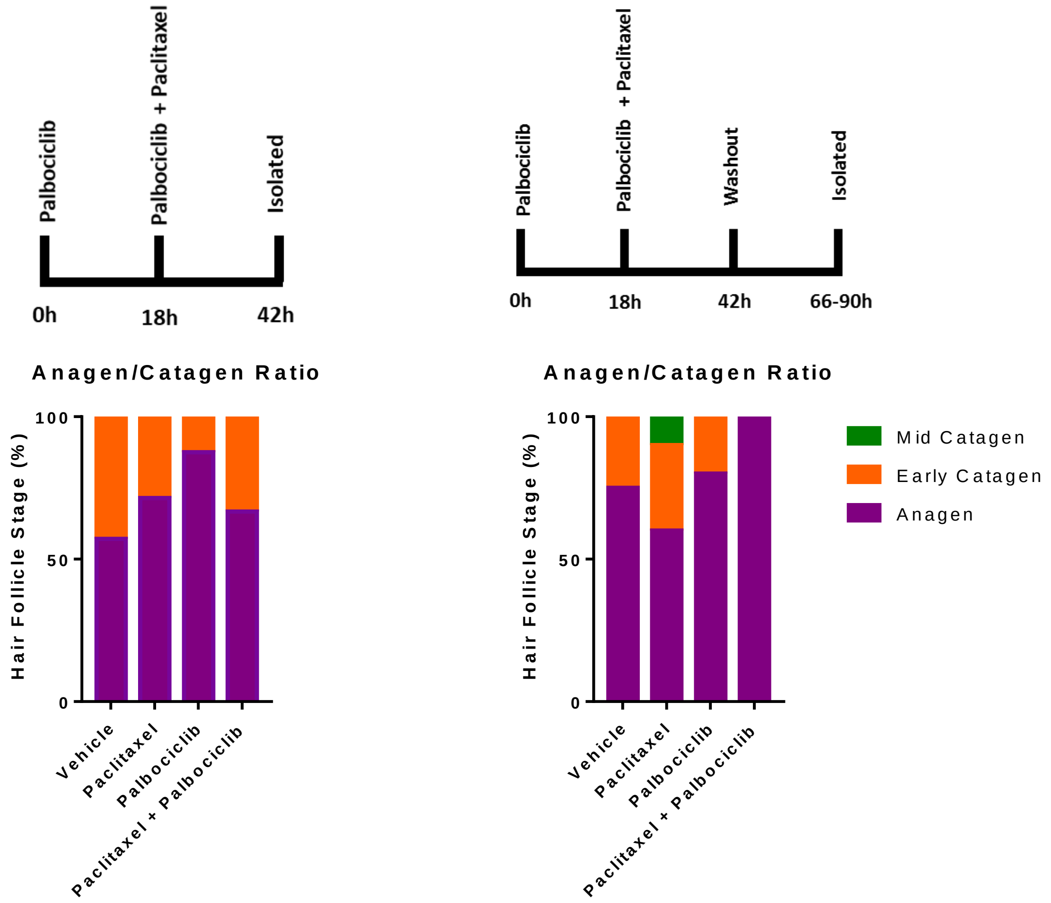

Figure S5 – Hair cycle analysis following palbociclib and/or paclitaxel treatment

Hair cycle staging analysis on hair follicles subject to the defined experimental conditions shows that neither paclitaxel or palbociclib increase the ratio of catagen versus anagen follicles. Each graph represents staging data pooled from 3 unique patient organ culture experiments.

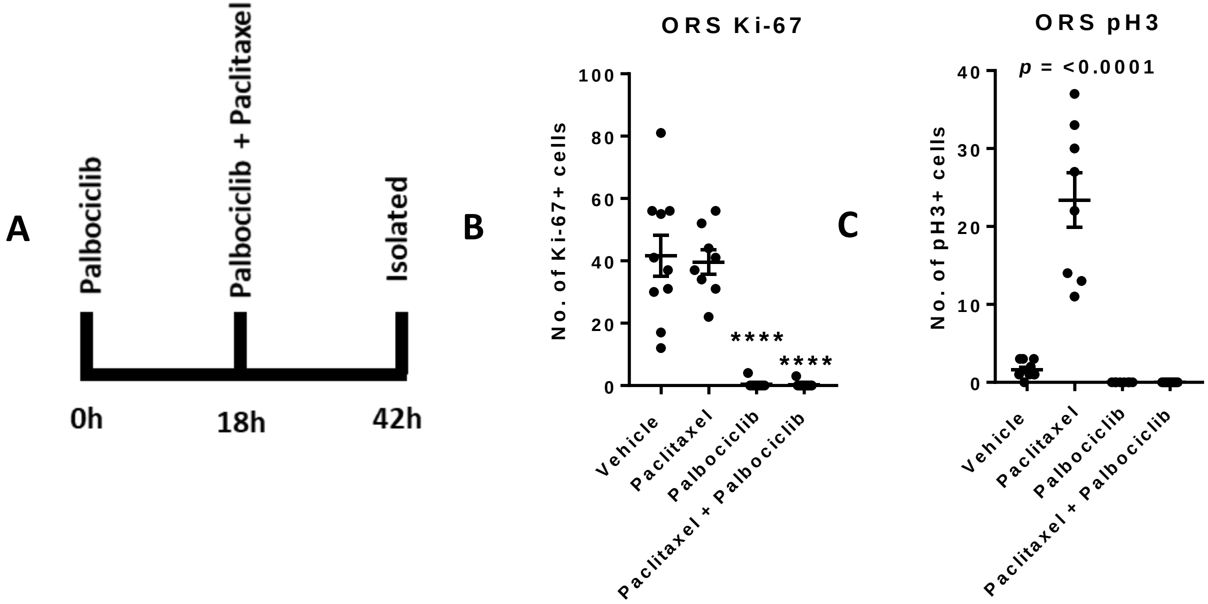

Figure S6 – Palbociclib blocks paclitaxel induced accumulation of pH3+ cells in the human hair follicle lower outer root sheath

A) Schematic of experimental design. Hair follicles (HFs) were pre-incubated with the CDK4/6 inhibitor palbociclib for 18h, followed by a further incubation period with and without paclitaxel (or paclitaxel alone) for an additional 24h.

B) HFs treated with palbociclib-only and palbociclib plus paclitaxel showed a significant (adjusted *p* values = 0.0001[****]) decrease in the number of Ki-67+ cells in the outer root sheath (ORS) in accordance with hair matrix data (see **Figure 5b**, main text). The number of Ki-67+ cells was unaffected by paclitaxel treatment, in accordance with data in **Figure 1a;5b** (main text**).** Ordinary one-way ANOVA with multiple comparisons performed using *N* of 9-11 HFs per condition from 2 patients.

C) Paclitaxel treatment significantly (adjusted *p* value = 0.0001[****]) increases the number of pH3+ cells in the ORS in accordance with hair matrix data (see **Figure 5C**, main text). This effect was not observed in the ORS when paclitaxel treated HFs were pre- and co-treated with palbociclib, as found in the hair matrix (see **Figure 5C**, main text). Ordinary one-way ANOVA with multiple comparisons performed using *N* of 8-11 HFs per condition from 2 patients.

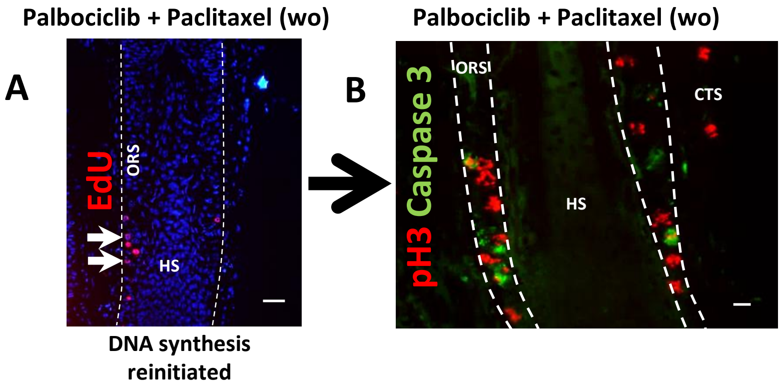

Figure S7 – Hair follicles that show resumed DNA synthesis in the outer root sheath following palbociclib drug washout show an accumulation of pH3+ cells in response to paclitaxel

In most hair follicles proliferation did not resume in the outer root sheath following washout of palbociclib (main text, **Figure 7**) which was in contrast to the hair matrix (main text, **Figure 6**). In the few hair follicles that did show resumed proliferation in the ORS **(A)** , this was met with an accumulation of pH3+ (and cleaved caspase 3+) cells induced by paclitaxel **(B)**. A – 50 µm scale; B - 20 µm scale. wo – washout.
