# Supplementary figures and images for: CDK4/6 inhibition mitigates stem cell damage in a novel model for taxane‐induced alopecia
Source: EMBO Mol Med. 2019 Sep 12;11(10):e11031. doi: 10.15252/emmm.201911031 (PMC6783643; doi:10.15252/emmm.201911031)

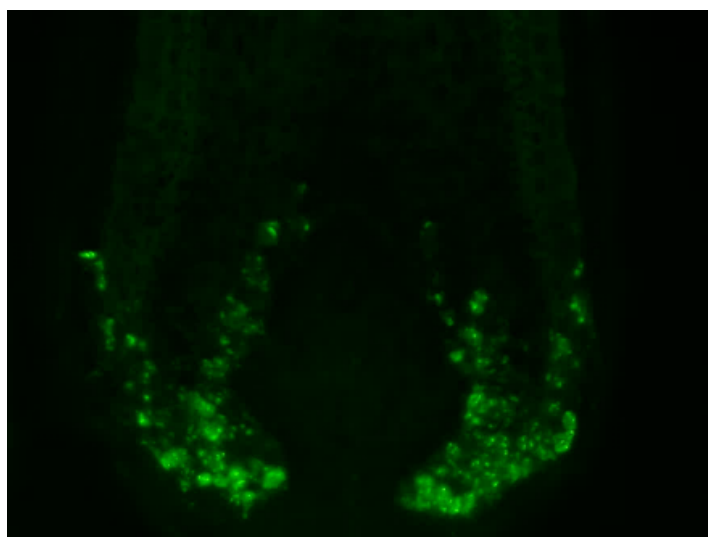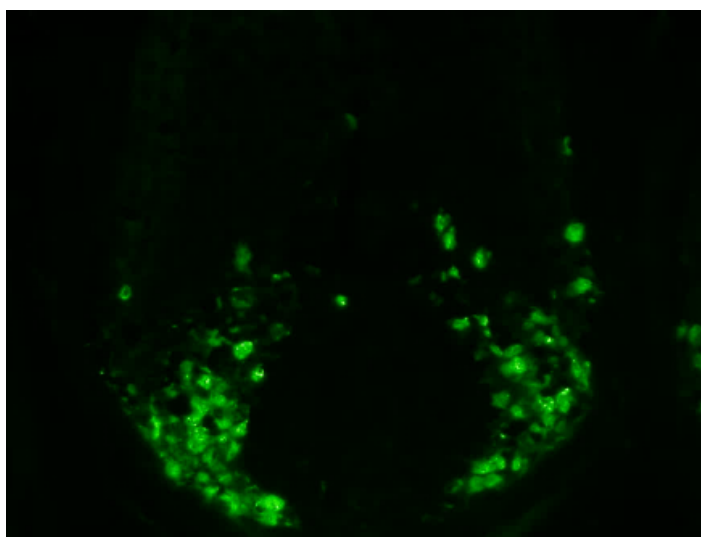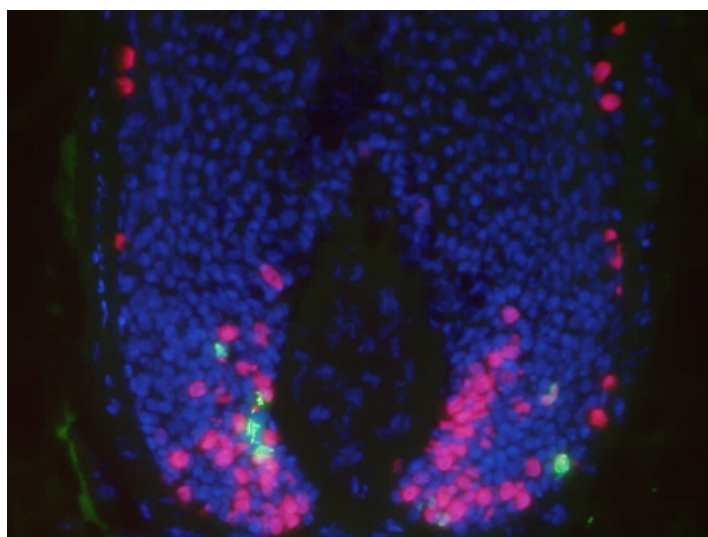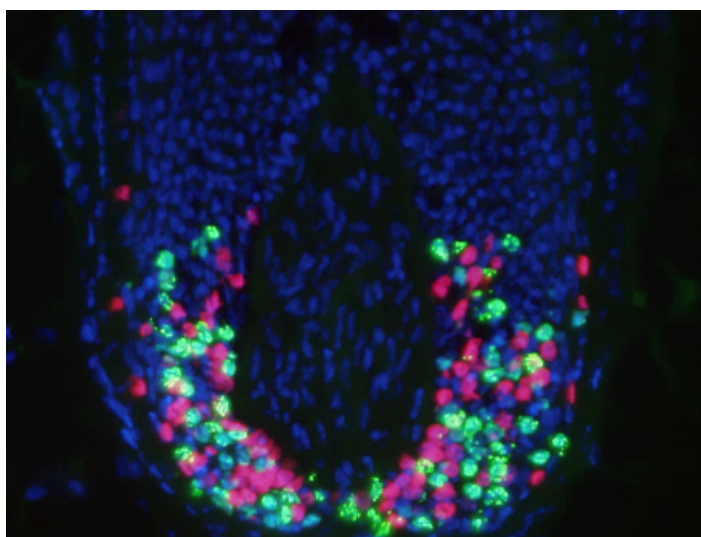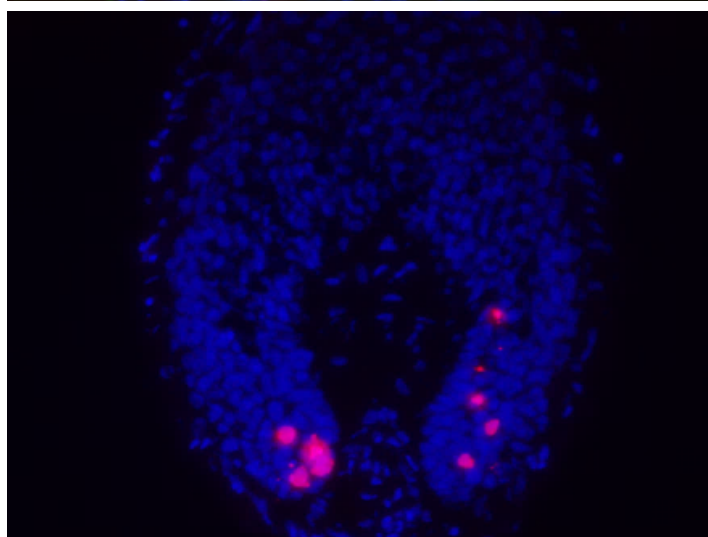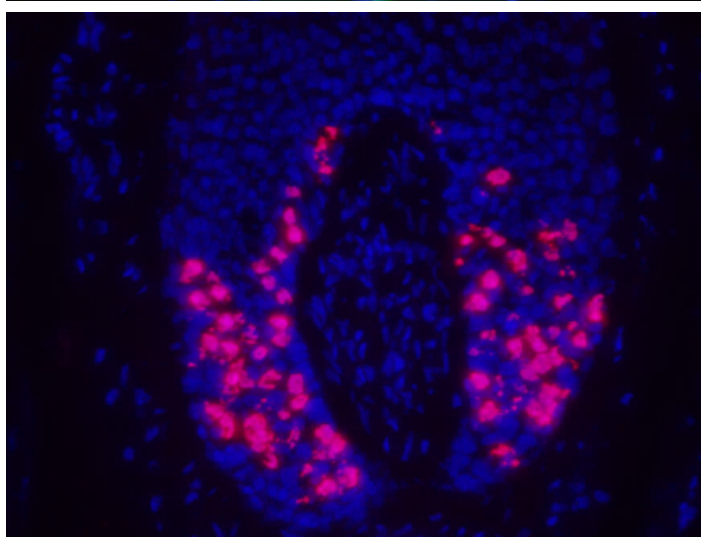

Supplement: Supplementary file 3 — Source Data for Figure 1 [file EMMM-11-e11031-s002.pdf]

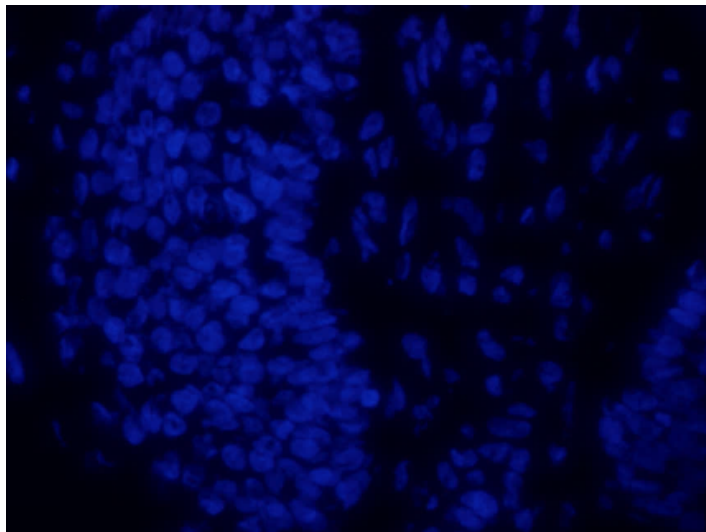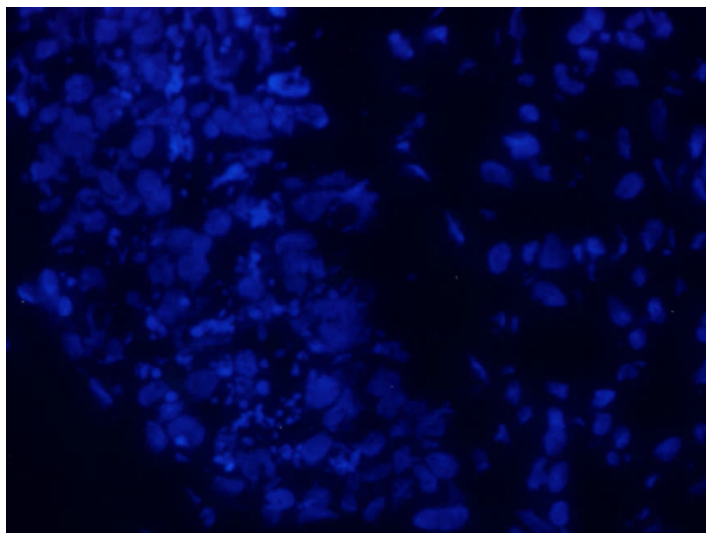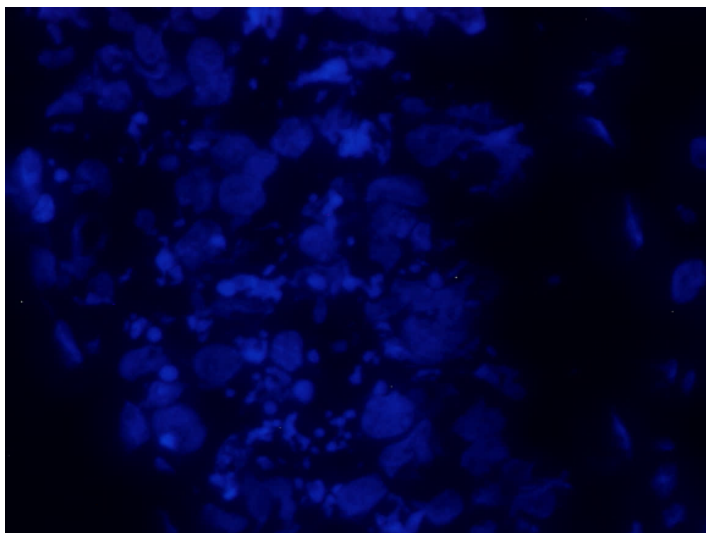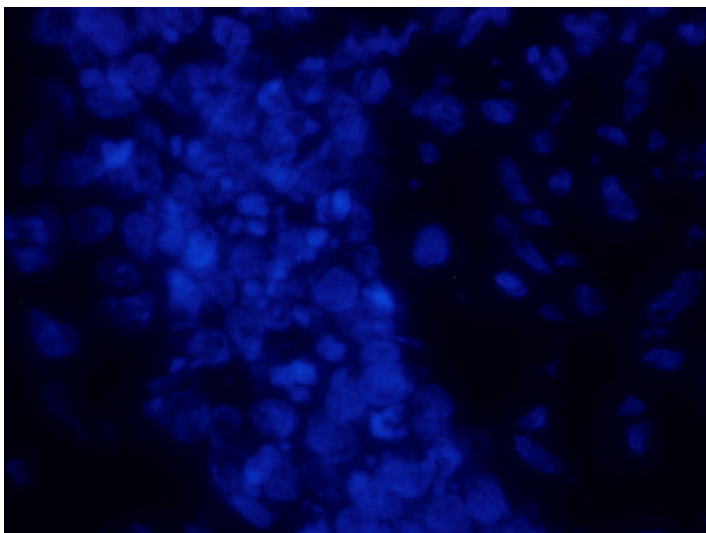

Supplement: Supplementary file 4 — Source Data for Figure 2 [file EMMM-11-e11031-s003.pdf]

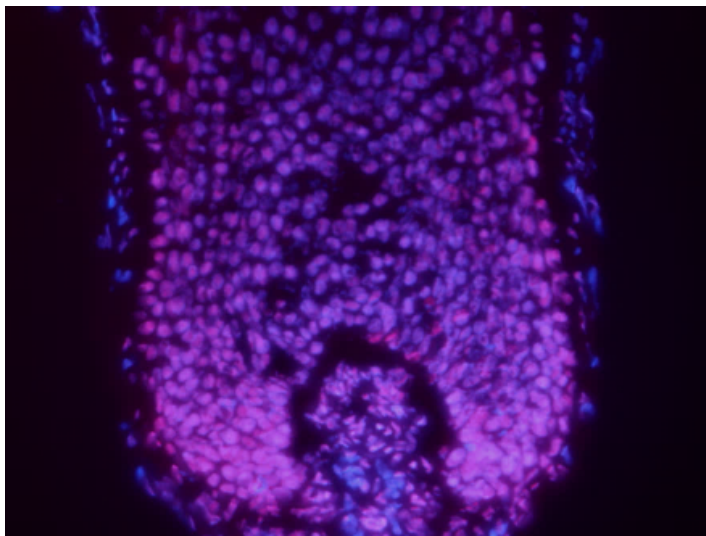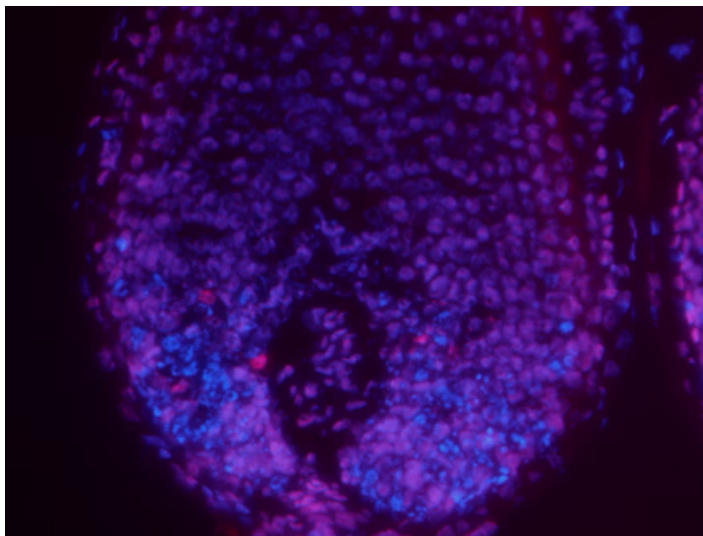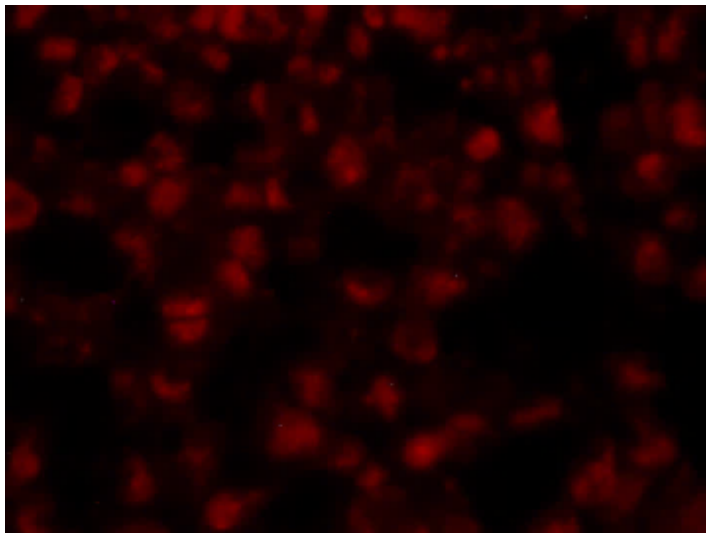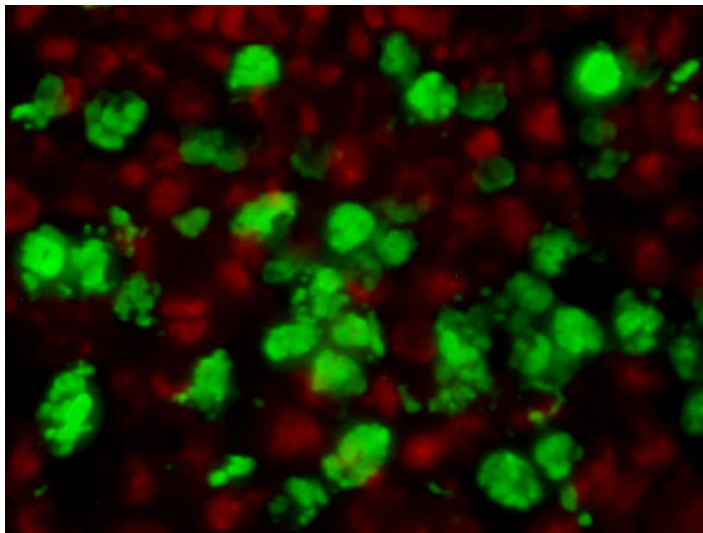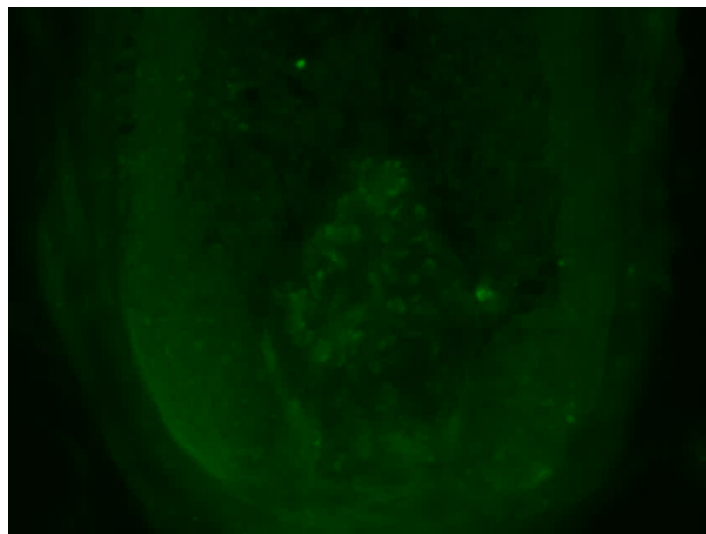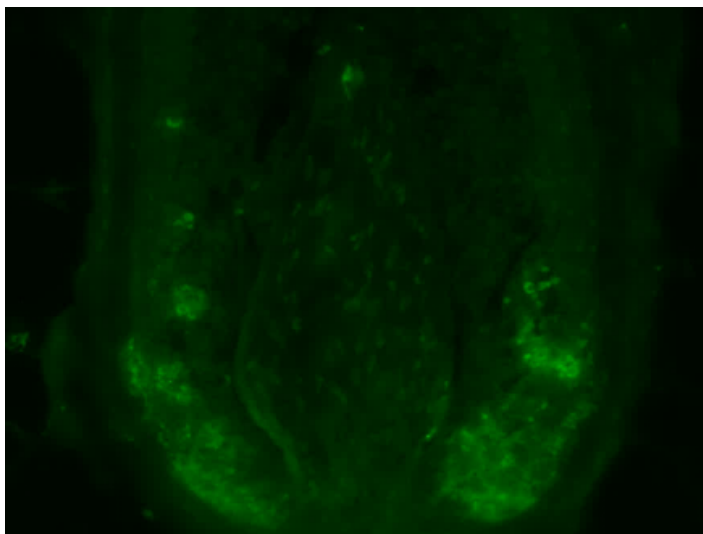

Supplement: Supplementary file 5 — Source Data for Figure 3 [file EMMM-11-e11031-s004.pdf]

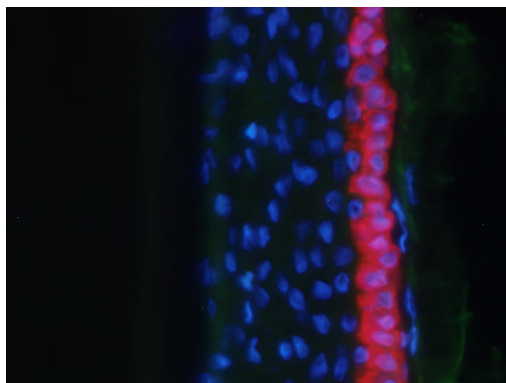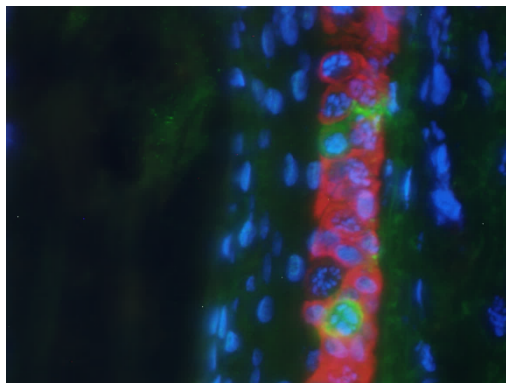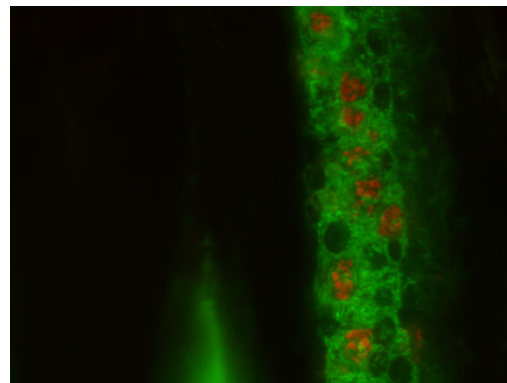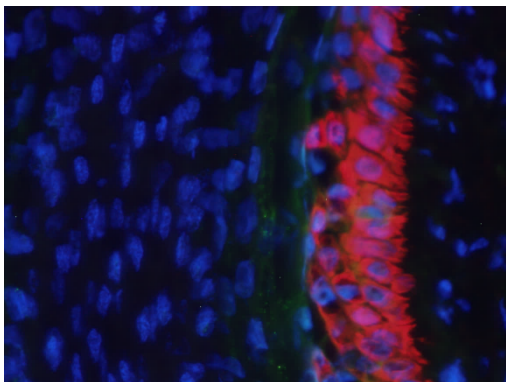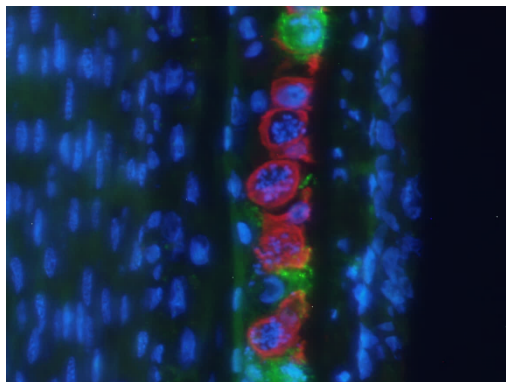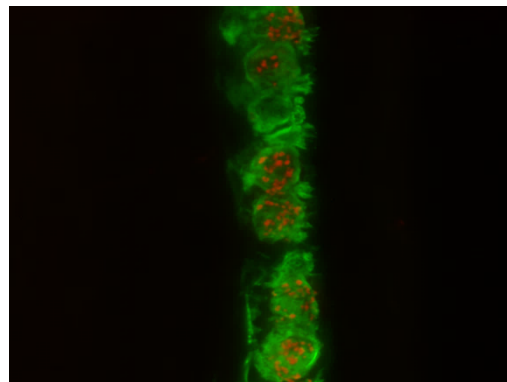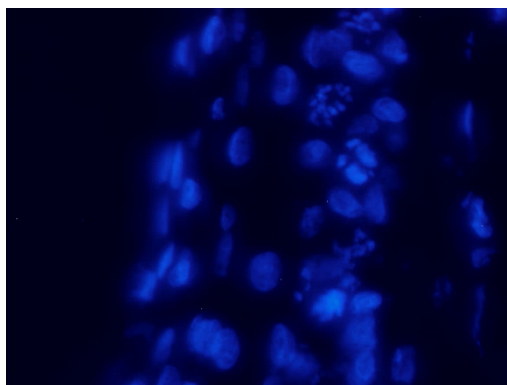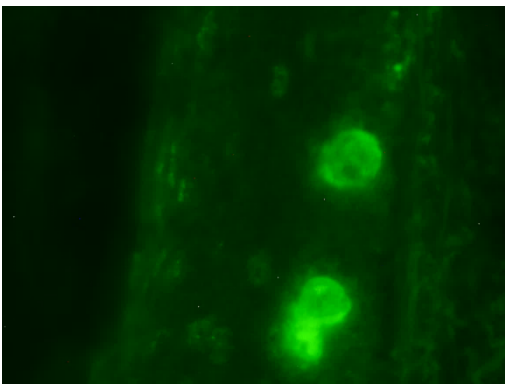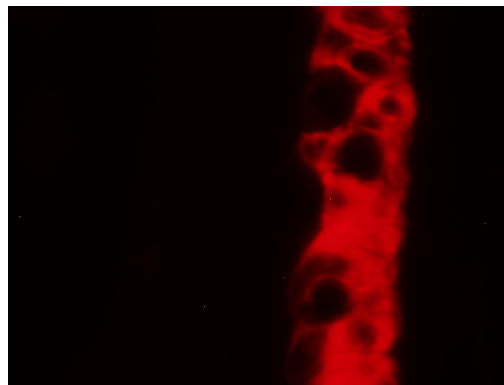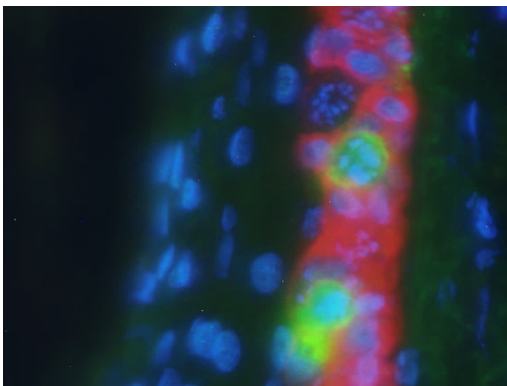

Supplement: Supplementary file 6 — Source Data for Figure 4 [file EMMM-11-e11031-s005.zip › emmm201911031-sup-0005-SDataFig4/emmm201911031-sup-0005-SDataFig4A.pdf]

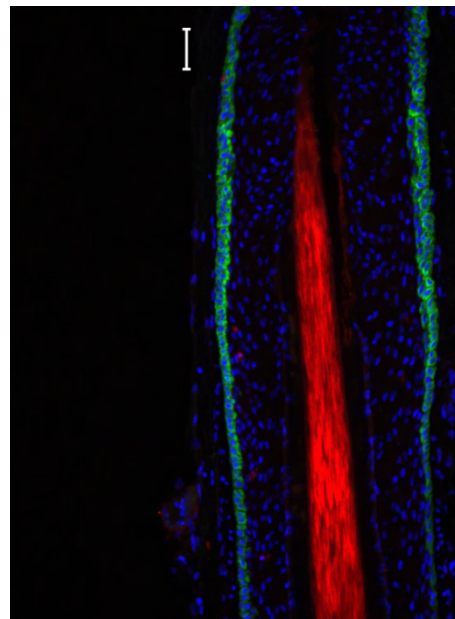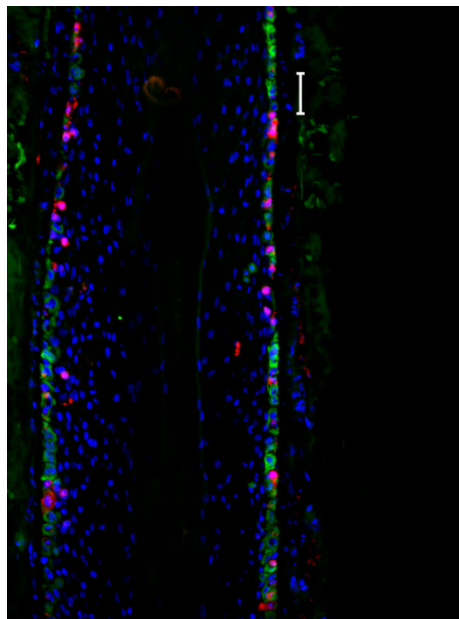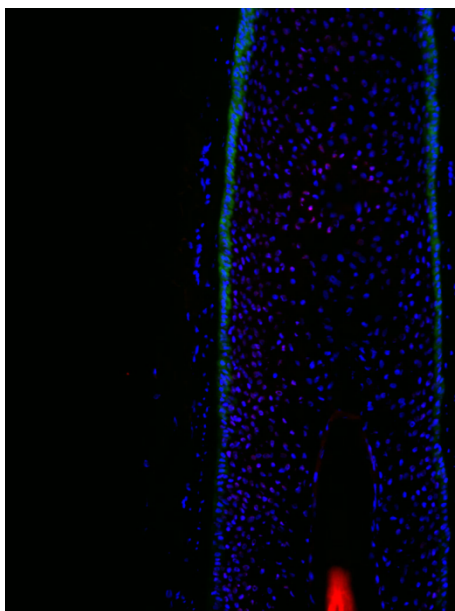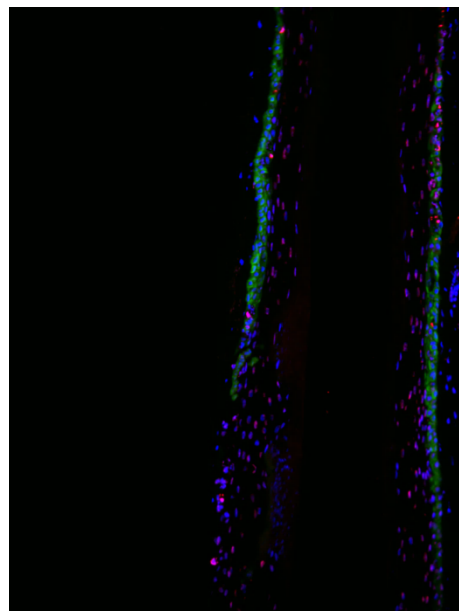

Supplement: Supplementary file 6 — Source Data for Figure 4 [file EMMM-11-e11031-s005.zip › emmm201911031-sup-0005-SDataFig4/emmm201911031-sup-0006-SDataFig4B-4E.pdf]

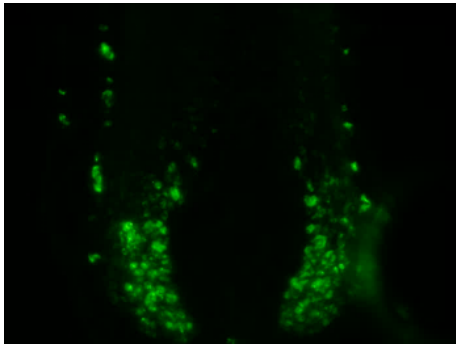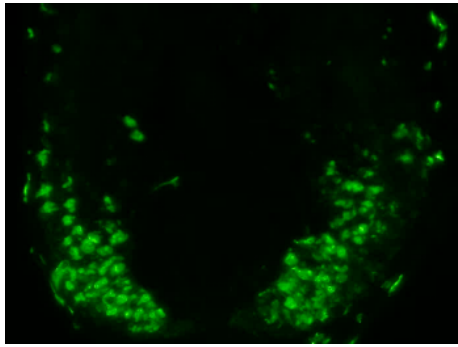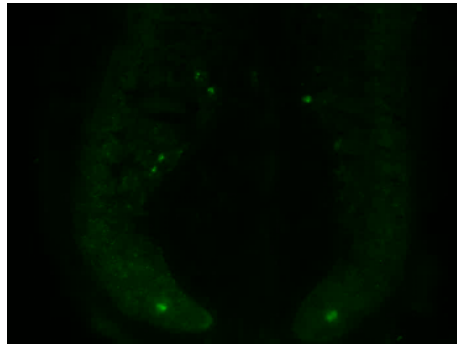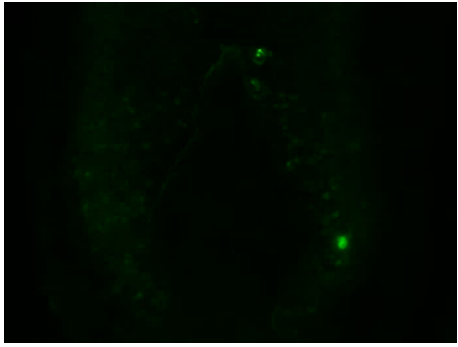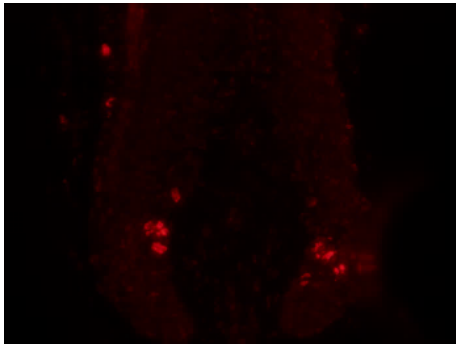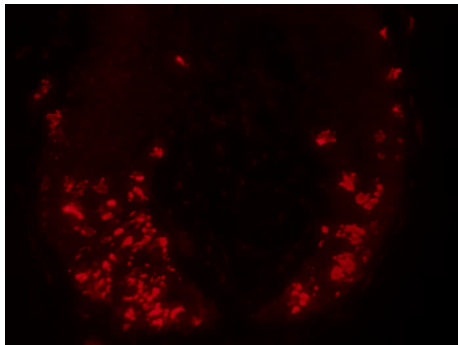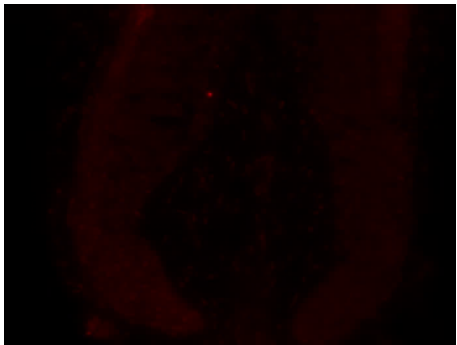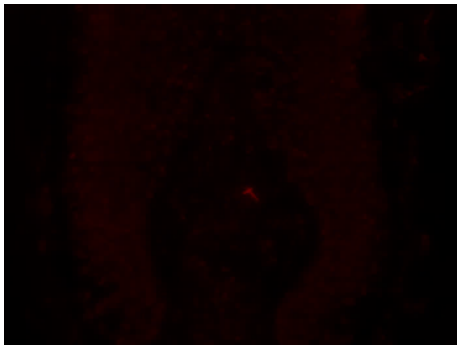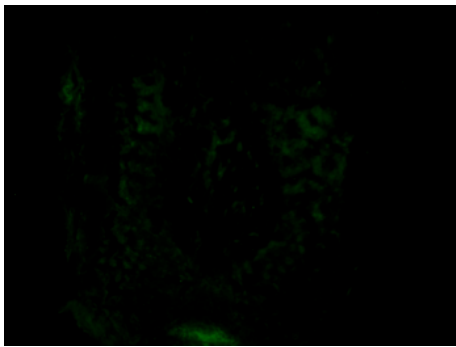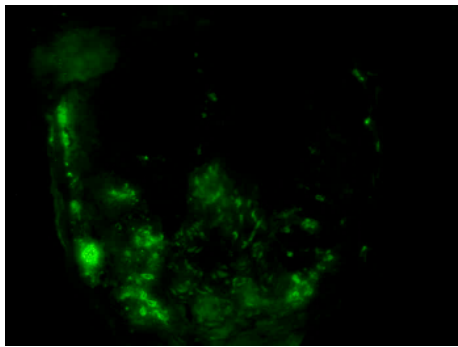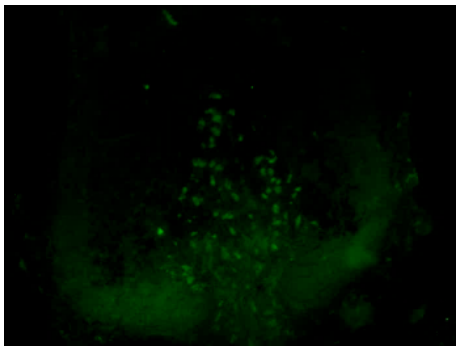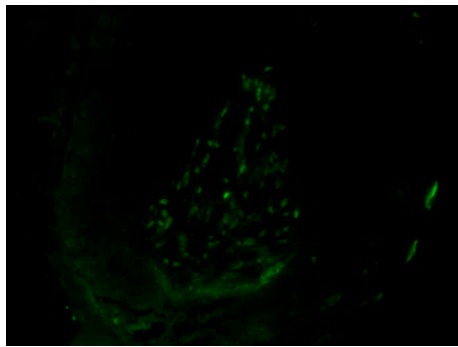

Supplement: Supplementary file 7 — Source Data for Figure 5 [file EMMM-11-e11031-s006.pdf]

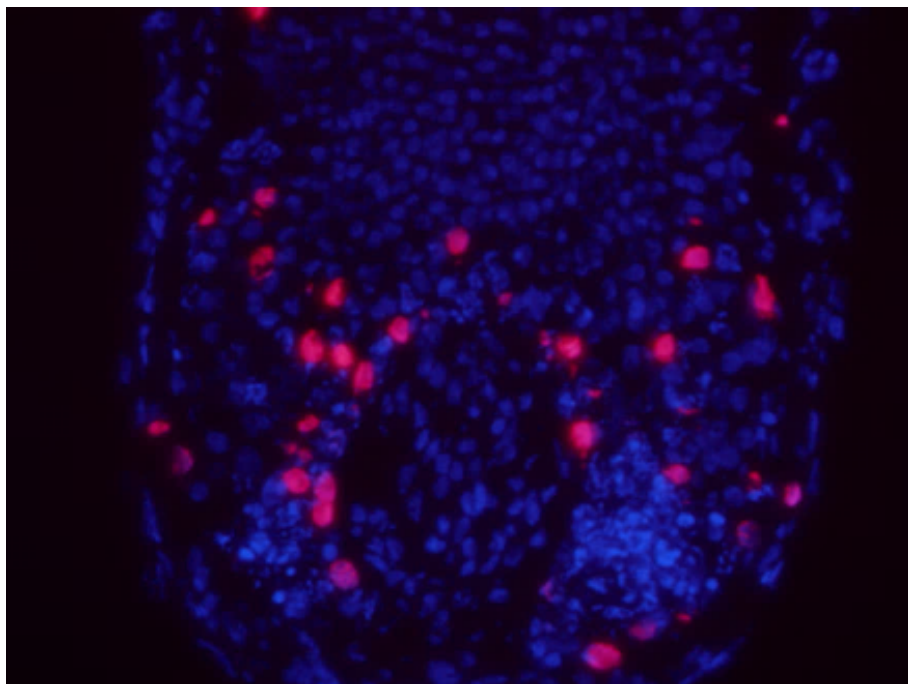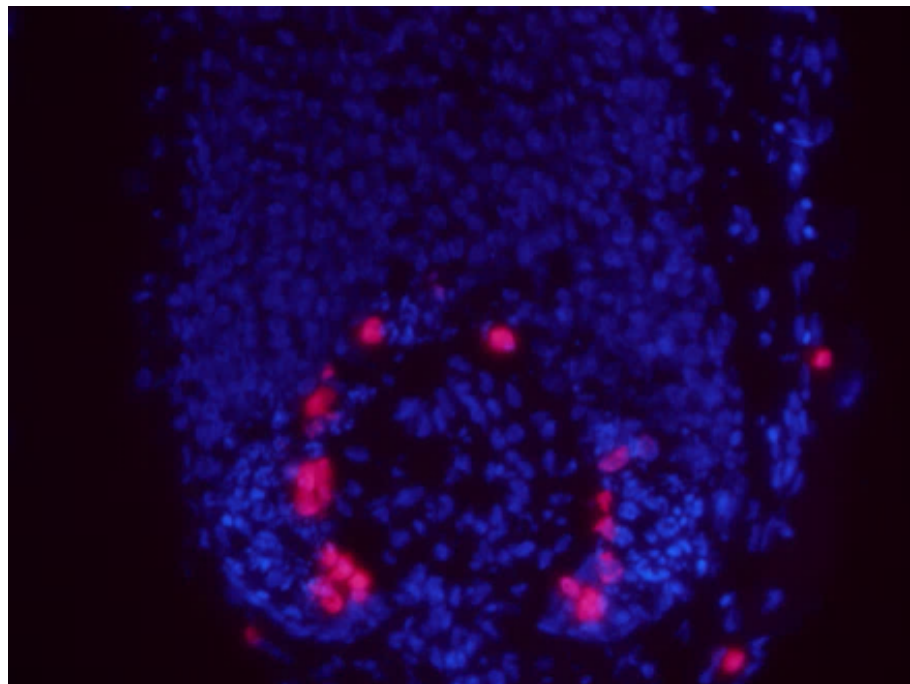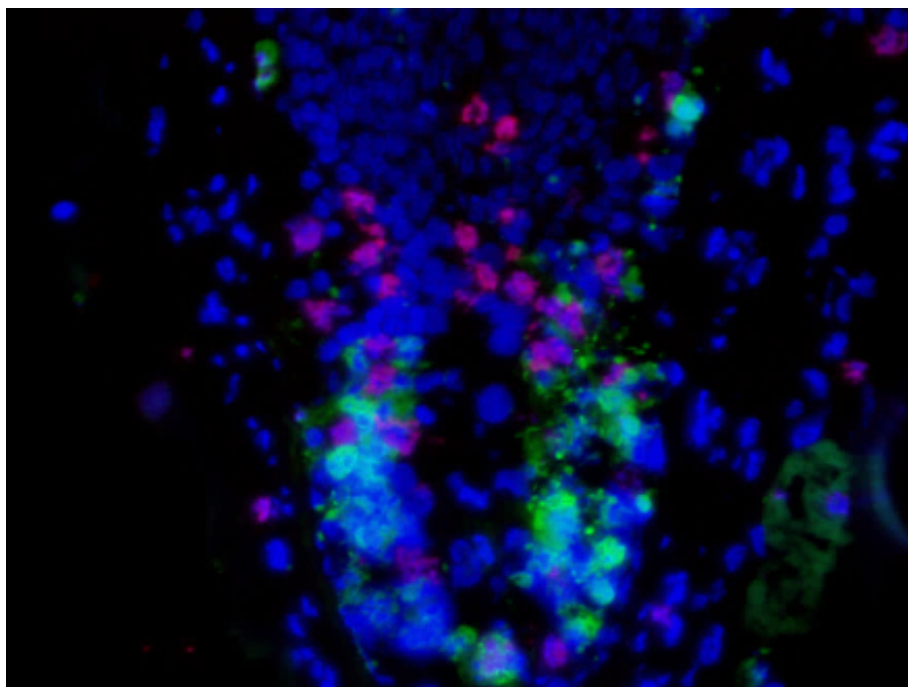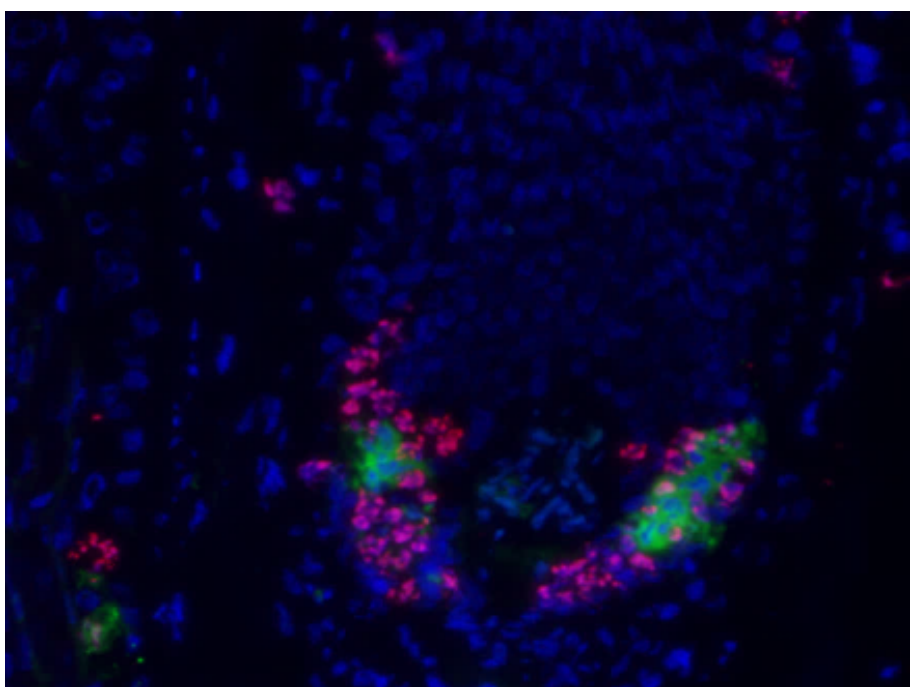

Supplement: Supplementary file 8 — Source Data for Figure 6 [file EMMM-11-e11031-s007.pdf]

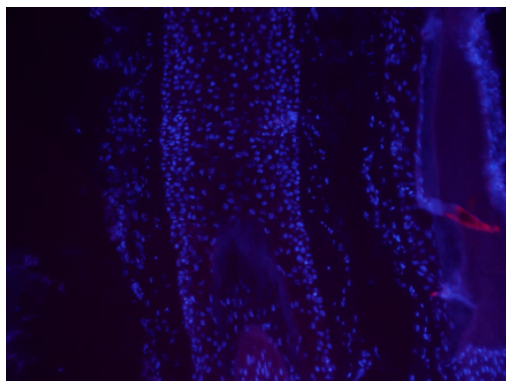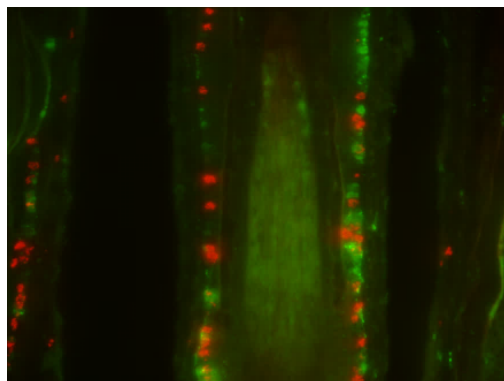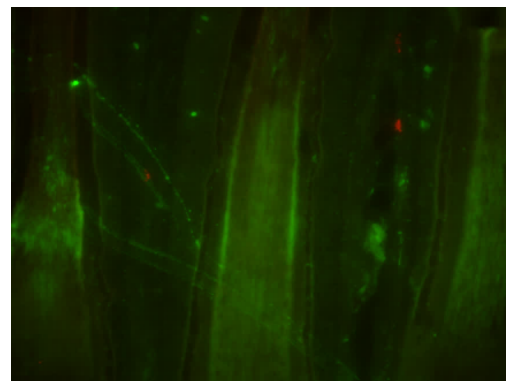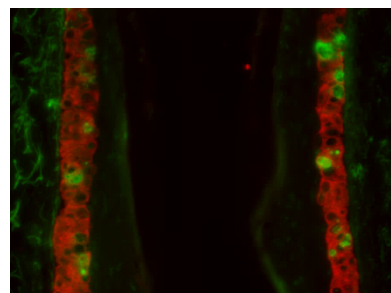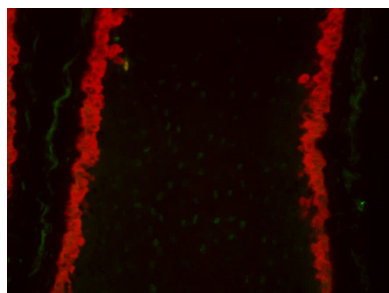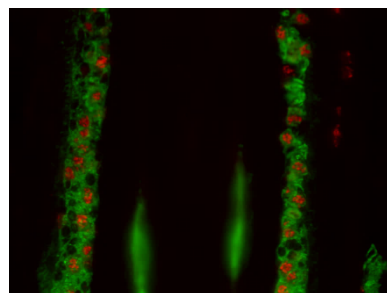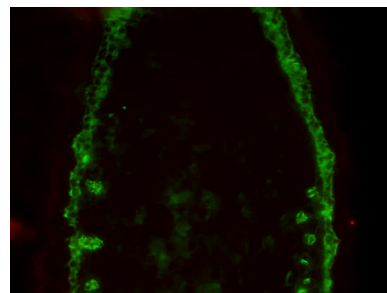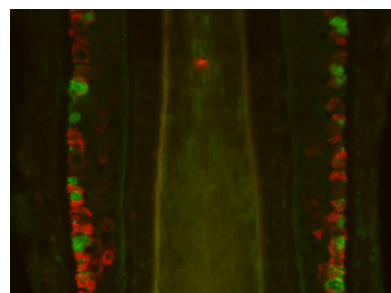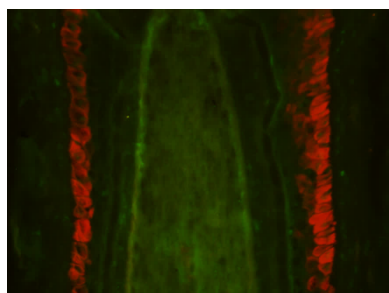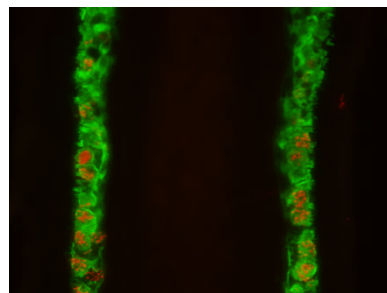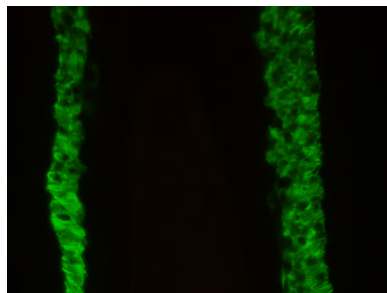

Supplement: Supplementary file 9 — Source Data for Figure 7 [file EMMM-11-e11031-s008.pdf]
